# Characteristics of injury of the corticospinal tract and corticoreticular pathway in hemiparetic patients with putaminal hemorrhage

**DOI:** 10.1186/1471-2377-14-121

**Published:** 2014-06-06

**Authors:** Jin Sun Yoo, Byung Yeon Choi, Chul Hoon Chang, Young Jin Jung, Seong Ho Kim, Sung Ho Jang

**Affiliations:** 1 Department of Physical Medicine and Rehabilitation, College of Medicine, Yeungnam University, 317-1, Daemyungdong, Namku, Taegu 705-717, Republic of Korea; 2 Department of Neurosurgery, College of Medicine Yeungnam University, Daegu, Republic of Korea

**Keywords:** Putaminal hemorrhage, Corticospinal tract, Corticoreticular pathway, Diffusion tensor imaging, Motor function

## Abstract

**Background:**

No study on the characteristics of injury of the corticospinal tract (CST) or corticoreticular pathway (CRP) in patients with putaminal hemorrhage has been reported. In this study, using diffusion tensor tractography, we attempted to investigate the characteristics of injury of the CST and CRP in hemiparetic patients with putaminal hemorrhage.

**Method:**

Fifty seven consecutive patients with putaminal hemorrhage and 57 healthy control subjects were recruited for this study. Diffusion tensor imaging was performed during the early period (8 ~ 30 days) after onset. We defined injury of the CST or CRP in terms of the configuration (discontinuation of a neural tract) or abnormal DTT parameters (the fractional anisotrophy value or fiber number was more than two standard deviations lower than that of normal control subjects). The Motricity Index, the modified Brunnstrom Classification, and the Functional Ambulation Categories were used for evaluation of motor function.

**Results:**

Among 57 patients, injury of the CST was found in 41 patients (71.9%) and injury of the CRP was found in 50 patients (87.8%), respectively, and 37 patients (64.9%) had injury of both the CST and CRP. All three motor functions of patients with injury of both the CST and CRP were significantly lower than those of patients with injury of either the CST or CRP (*p* < 0.05).

**Conclusion:**

Our results indicate that the putaminal hemorrhage frequently accompanies injury of both the CST and CRP, and the CRP appears to be more vulnerable to putaminal hemorrhage than the CST. These findings suggest the necessity for evaluation of both the CRP and the CST in patients with putaminal hemorrhage.

## Background

Spontaneous intracerebral hemorrhage (ICH), comprising 48 ~ 67% of all spontaneous ICH, most commonly occurs in the putamen [1]. Various neural tracts, particularly the neural tracts for motor function, including the corticospinal tract (CST) and corticoreticular pathway (CRP), are located near the putamen, consequently, motor weakness is one of the most common neurological manifestations in patients with putaminal hemorrhage [2-5]. Therefore, clarification of the cause of motor weakness is essential for prognosis and for adoption of scientific strategies for successful rehabilitation [6-11]. However, little is known about this topic.

In the human brain, the neural tracts for motor function are classified according to the CST and the non-CST. The CST and the corticoreticulospinal tract have been regarded as important neural tracts for mediation of voluntary movements [12,13]. The main function of the CST is control of movements of distal extremities, particularly fine-motor movements of the hand [12-14], while the corticoreticulospinal tract, consisting of the corticoreticular pathway (CRP) and the reticulospinal tract, innervates proximal extremities and axial muscles [9,15-18]. Therefore, elucidation of the state of the CST and CRP is important for determination of the cause of motor weakness in stroke patients.

Conventional neuroimaging techniques, such as brain CT and MRI have limitations in accurate assessment of neural tracts, including the CST and CRP [19]. By contrast, recently developed diffusion tensor tractography (DTT), derived from diffusion tensor imaging (DTI), enables three-dimensional reconstruction and estimation of the CST and CRP [2,3,8-10,16,20-25]. Consequently, many studies have reported on injury of the CST or CRP in stroke patients with motor weakness [8-11,26]. However, no study on the characteristics of injury of the CST or CRP in patients with putaminal hemorrhage has been reported so far.

In this study, using DTT, we attempted to investigate the characteristics of injury of the CST and CRP in hemiparetic patients with putaminal hemorrhage.

## Methods

### Subjects

Among patients admitted for rehabilitation to the rehabilitation department of a university hospital, 57 consecutive patients (37 males, 20 female; mean age 55.1 years, range 34 ~ 74, right ICH 31, left ICH 26) and 57 age- and sex-matched healthy control subjects (36males, 21 females; mean age 52.6 years, range 33 ~ 67) with no history of neurological or psychiatric problems were recruited for this study. The 57 patients were recruited according to the following criteria: (1) first-ever stroke, (2) age: 20 ~ 75 years, (3) DTI scanning performed at the early stage after ICH: between eight and 30 days after onset, (4) spontaneous putaminal ICH, confirmed by a neuroradiologist, (5) motor weakness in the contralateral extremities of ICH, and (6) no history of neurologic, psychiatric, or traumatic brain disease. This retrospective study was approved by the Institutional Review Board of Yeungnam university hospital.

### Clinical evaluation

Motor function was evaluated at the time of DTI scanning, using the Motricity Index (MI), the modified Brunnstrom Classification (MBC), and the Functional Ambulation Categories (FAC) [27-29]. The MI score is a modification of the Medical Research Council scoring system, with a maximum score of 100 [27]. The MBC score is as follows: 1; unable to move fingers voluntarily, 2; able to move fingers voluntarily, 3; able to close hand voluntarily, unable to open hand, 4; able to grasp a card between the thumb and medial side of the index finger, able to extend fingers slightly, 5; able to pick up and hold a glass, able to extend fingers, 6; able to catch and throw a ball in a near-normal fashion, able to button and unbutton a shirt [28]. The FAC was designed for characterization of levels of assistance required during a 15 m walk. The six categories include the following: 0; non-ambulatory, 1; need for continuous support from one person, 2; need for intermittent support from one person, 3; need verbal supervision only, 4; help required on stairs and uneven surfaces, and 5; ability to walk independently anywhere [29]. The reliability and validity of MRC, MI, MBC, and FAC are well established [27-29].

### DTI acquisition and analysis

**Figure 1:**
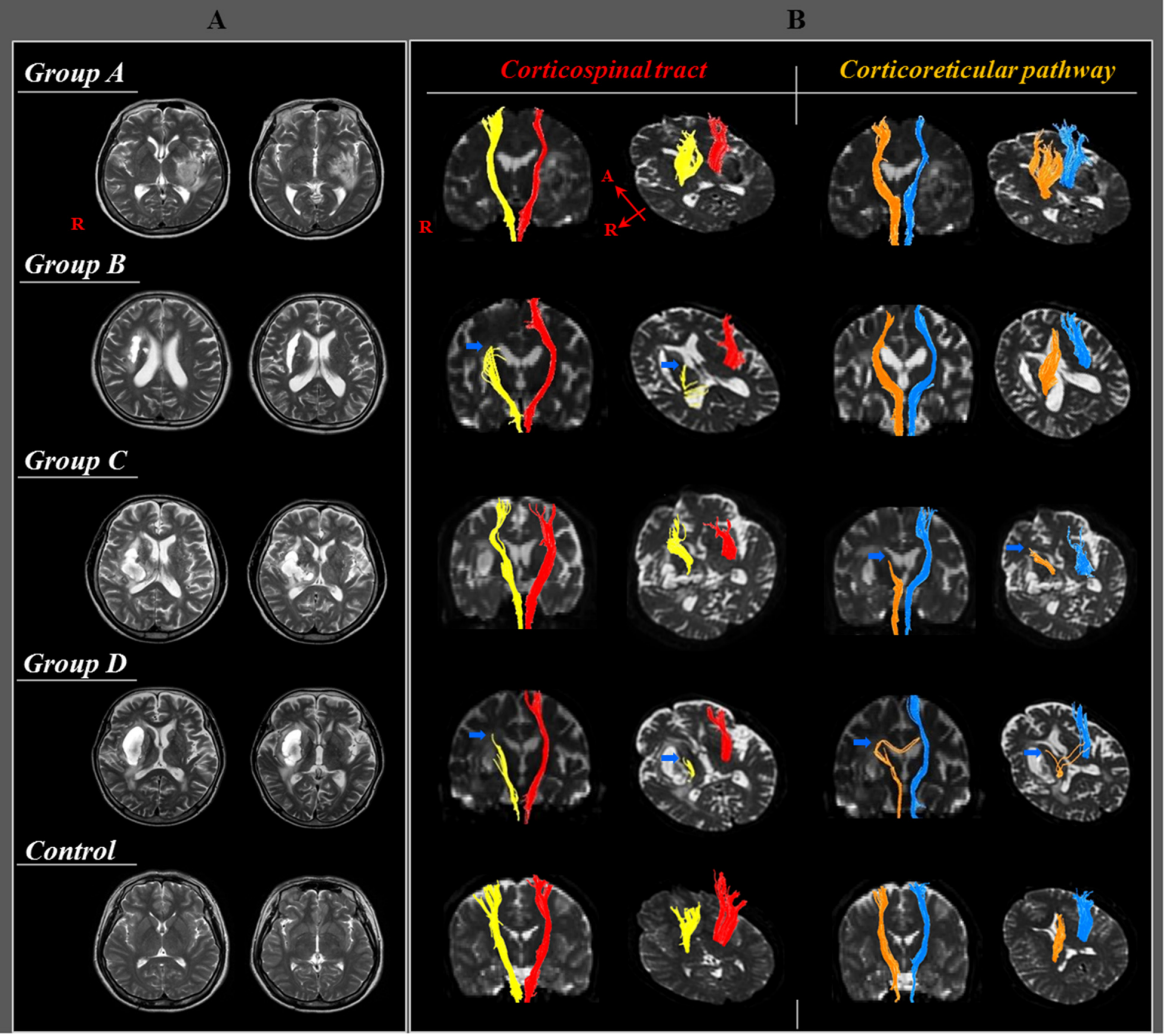
T2-weighted brain MR images (A) and diffusion tensor tractography image (B) of the corticospinal tract and corticoreticular pathway for each patient of four groups and a control subject (arrow: discontinuation of a neural tract).

DTI data were acquired at the beginning of rehabilitation (average 17 days, range: 8–30 days after onset) using a 1.5-T Philips Gyroscan Intera system equipped with a synergy-L Sensitivity Encoding (SENSE) head coil utilizing a single-shot, spin-echo planar imaging pulse sequence. For each of the 32 noncollinear and noncoplanar diffusion-sensitizing gradients, we acquired 67 contiguous slices parallel to the anterior commissure-posterior commissure line in order to avoid sphenoidal susceptibility artifact. Imaging parameters were as follows: matrix = 128 × 128 matrix, field of view = 221 × 221 mm^2^, TR = 10,726 ms, TE = 76 ms, SENSE factor = 2, EPI factor = 67 and b = 1000 s/mm^2^, NEX = 1, and slice thickness = 2.3 mm. Eddy current-induced image distortions were removed using affine multiscale two-dimensional registration at the Oxford Centre for Functional Magnetic Resonance Imaging of Brain (FMRIB) Software Library. DTI-Studio software (CMRM, Johns Hopkins Medical Institute, Baltimore, Md., USA) was used for reconstruction of the CST and CRP. For the CST, a seed ROI was drawn on the portion of the CST in the anterior mid-pons on a 2D fractional anisotropy (FA) color map, and a target ROI was drawn on the portion of the CST in the anterior lower pons [30]. For the CRP, a seed ROI was placed on the reticular formation of the medulla, and a target ROI was placed on the midbrain tegmentum. Fiber tracts passing through both ROIs were designated as final tracts of interest [3,17]. Termination criteria used for fiber tracking included FA <0.2 and an angle change of >60 [31]. The FA value and fiber number of the CST and CRP in the affected hemisphere were estimated. We defined injury of the CST or CRP in terms of the configuration (discontinuation of a neural tract at or around the hematoma) or abnormal DTT parameters (the FA value or fiber number was more than two standard deviations lower than that of normal control subjects). We classified the findings of the CST and CRP according to three types, respectively: type I: normal configuration and DTT parameters, type II: normal configuration and abnormal DTT parameters, and type III: abnormal configuration and DTT parameters. The patients were classified into four groups according to injury of the CST or CRP: group A: patients with intact CST and CRP, group B: patients with injured CST and intact CRP, group C: patients with intact CST and injured CRP, and group D: patients with injured CST and CRP (Figure 1).

The volume of hematoma was calculated on T2-weighted MRI images using a picture archiving communication system (PACS, Marotech, Korea) at the time of DTI scanning. We measured maximum width (X), length (Y), and height (Z) of ICHs at the level where hemorrhage in the putamen was definite [32]. The volume of hematoma was calculated according to the formula:

$$\begin{array}{l} \mathrm{ICH}\;\mathrm{volume}\left(\mathrm{mV}\right)\;=\;4/3\times 1/16\times \pi \times X\left(\mathrm{cm}\right)\\ \;\times Y\left(\mathrm{cm}\right)\times Z\left(\mathrm{cm}\right). \end{array}$$

### Statistical analysis

Statistical analysis was performed using SPSS 17.0 for Windows (SPSS Inc., Chicago, Ill., USA). The Kruskal-Wallis test with the Mann–Whitney U post hoc test was used to determine the significance of ICH volume, MI, MBC, and FAC in the four groups and statistical significance was accepted for *p* < 0.05. Correlations between the ICH volume and MI, MBC, and FAC were determined using Spearman correlation test, and statistical significance was accepted for *p* < 0.05.

## Results

A summary of the demographic and clinical data of the patient group is shown in Table 1. Of the 57 patients, three patients (5.3%) belonged to group A, four patients (7.0%) belonged to group B, 13 patients (22.8%) belonged to group C, and 37 patients (64.9%) belonged to group D. Significantly lower MI, MBC, and FAC was observed for group D, compared with groups A, B, and C (*p* < 0.05). No differences in MI, MBC, and FAC were observed between group B and group C (*p* > 0.05). Higher MI was observed for group A than for groups B and C (*p* < 0.05). However, no significant differences in MBC and FAC were observed for group A compared with groups B and C (*p* > 0.05). No significant difference in mean age and ICH volume was observed among the four groups (*p* > 0.05). In addition, no correlation was found between the ICH volume, and MI, MBC, and FAC (*p* > 0.05).

**Table 1:** Demographic and clinical data of the patient group

	**Group A**	**Group B**	**Group C**	**Group D**
Number of patients, n (%)	3 (5.3%)	4 (7.0%)	13 (22.8%)	37 (64.9%)
Mean age, years	61.0 ± 5.6	50.0 ± 16.5	60.8 ± 10.7	53.3 ± 10.5
Hematoma volume, mV	7.8 ± 0.4	7.5 ± 1.3	9.1 ± 7.1	10.8 ± 6.1
MI	74.6 ± 1.4	64.3 ± 6.9	66.3 ± 7.3	35.5 ± 20.8
MBC	5.7 ± 0.6	4.0 ± 1.4	5.0 ± 0.8	2.2 ± 0.9
FAC	4.0 ± 0.0	2.8 ± 0.9	2.5 ± 1.8	0.6 ± 0.9

MI: Motricity index.

MBC: modified Brunnstrom classification.

FAC: functional ambulation category.

Values indicate mean ± standard deviation.

Table 2 shows the distribution of patients according to the type of injury of the CST and CRP in four groups. Among 57 patients, injury of the CST was found in 41 patients (71.9%) and injury of the CRP was found in 50 patients (87.8%). The proportion of patients with type II was the same (17.6%) for injuries of both the CST and the CRP; however, the proportion of type III was higher in patients with injury of the CRP (70.2%) than in those with injury of the CST (54.3%).

**Table 2:** Distribution according to the type of injury of the corticospinal tract and corticoreticular pathway

	**CST**			**CRP**		
**Group**	**Type I**	**Type II**	**Type III**	**Type I**	**Type II**	**Type III**
A	3 (5.3%)	-	-	3 (5.3%)	-	-
B	-	3 (5.3%)	1 (1.7%)	4 (7.0%)	-	-
C	13 (22.8%)	-	-	-	3 (5.3%)	10 (17.6%)
D	-	7 (12.3%)	30 (52.6%)	-	7 (12.3%)	30 (52.6%)
Total number	16 (28.1%)	10 (17.6%)	31 (54.3%)	7 (12.3%)	10 (17.6%)	40 (70.2%)

CST: corticospinal tract.

CRP: corticoreticular pathway.

Values indicate the number of patients (%).

## Discussion

In the current study, using DTT, we examined the characteristics of injury of the CST and CRP in 57 consecutive hemiparetic patients with putaminal ICH. Our findings were as follows. First, the incidence of injury; group A – 5.3%, group B -7.0%, group C – 22.8%, and group D - 64.9%. Consequently, among the 57 consecutive patients with putaminal hemorrhage, injury of the CST was found in 41 patients (71.9%) and injury of the CRP was found in 50 patients (87.8%). These results indicate that the putaminal hemorrhage frequently accompanies injury of both the CST and CRP, and the CRP appears to be more vulnerable to putaminal hemorrhage than the CST. The greater vulnerability of the CRP appears to be ascribed to the anatomical characteristics of the CST and CRP; the CST and the CRP are located within close proximity to one another and the CRP is located closer to the center of the putamen than the CST in the anteroposterior direction [2,3]. However, conduct of further studies on this topic will be necessary. Second, the severity of injury of the CST and CRP; CST- type II (17.6%) and type III (54.3%); CRP- type II (17.6%) and type III (70.2%). The FA value is the most commonly used DTI parameter reflecting the degree of directionality of microstructures, such as axons, myelin, and microtubules, [33-35]. The fiber number reflects the total number of voxels in a neural tract [9]. Therefore, the decrement of FA value or of fiber number of a neural tract indicates an injury of the neural tract. Type II may indicate mild or partial injury because this type means the decrement of FA value or fiber number withpreservation of the integrity of a neural tract. By contrast, type III might suggest more severe or complete injury of a neural tract because this type indicates discontinuation of a neural tract. Therefore, our results suggest that in the case of an accompanying putaminal hemorrhage, regarding injury of the CRP, more patients showed more severe injury than that of the CST, although the proportion of mild injury of both neural tracts was the same. The third was the clinical characteristics of the four groups. The average values of three clinical scales were as follows, in order with better function: the MI (general motor function, full score: 100) was as follows: group A, group C, group B, and group D; the MBC (hand function, full score; 6); group A, group C, group B, and group D; the FAC (gait function, full score; 5); group A, group B, group C, and group D. Scores for all motor functions were worse in terms of general motor function, hand function, and gait in patients with injury of both the CST and CRP. Patients with injury of the CST, which is closely related to hand function, showed worse hand function than patients with injury of the CRP, and patients with injury of the CRP, which is more related to gait function, showed lower FAC value than patients with injury of the CST. However, no statistical differences in terms of hand and gait function were observed between the two groups. The fourth was the clinical correlation with the size of hematoma. No significant difference in the volume of hematoma was observed among the four groups and no correlation in terms of the MI, MBC, and FAC was found between ICH volume and all clinical scales. These results indicate that hematoma size is not important to injury of the CST or CRP. On the contrary, it is possible that the location of hematoma is more important in injury of the CST or CRP than the size of hematoma.

## Conclusions

In conclusion, we investigated the characteristics of injury of the CST and CRP in 57 hemiparetic patients with putaminal hemorrhage. According to our findings, 64.9% of patients had injury of both the CST and CRP, and the incidence (87.8%) of CRP injury was higher than that (71.9%) of CST injury. Our results indicate that an accompanying putaminal hemorrhage tends to occur in cases involving injury of both the CST and CRP, with greater vulnerability of the CRP than the CST. In addition, patients with injury of the CST and CRP showed worse motor function than patients with injury of either the CST or CRP. These results suggest the necessity for evaluation of both the CRP and the CST in patients with putaminal hemorrhage. Many studies have reported on injury of the CST or CRP in patients with putaminal hemorrhage using DTT [8-11,26]. However, this is the first study to investigate the characteristics of injury of the CST and CRP in a large number of consecutive patients with putaminal hemorrhage. However, some limitations should be considered in interpretation of this study. First, we recruited the patients among those with putaminal hemorrhage who had been admitted for rehabilitation. Therefore, it is possible that among all patients with putaminal hemorrhage, we recruited patients with severe clinical manifestations. Second, DTI may underestimate fiber tracts due to hematoma or peri-hematomal edema in patients with putaminal hemorrhage. In addition, fiber complexity and crossing fiber effect can prevent reflection of the fiber tracts [36-38]. Therefore, in order to overcome these limitations, conduct of further studies should be encouraged.

## Abbreviations

AF: Arcuate fasciculus; DTT: Diffusion tensor tractography; DTI: Diffusion tensor imaging; K-WAB: Korean-Western aphasia battery; K-MMSE: Korean version of the mini mental state examination; FMRIB: Functional magnetic resonance imaging of brain; FA: Fractional anisotropy; ADC: Apparent diffusion coefficient.

## Competing interests

The authors declare that we have no competing interests.

## Authors’ contributions

JSY participated in the design of the study, collection and analysis of data, and drafting the manuscript. BYC, CHC, JYJ, and SHK participated in the design of the study and collection of data. SHJ participated in the design of the study, funding, and writing the manuscript. All authors read and approved the final manuscript.

## Pre-publication history

The pre-publication history for this paper can be accessed here:

http://www.biomedcentral.com/1471-2377/14/121/prepub
